# Baseline (derived) neutrophil-lymphocyte ratio associated with survival in gastroesophageal junction or gastric cancer treated with ICIs

**DOI:** 10.3389/fonc.2025.1404695

**Published:** 2025-01-24

**Authors:** Chengyang Yu, Hao Jiang, Liezhi Wang, Zufu Jiang, Chong Jin

**Affiliations:** Department of General Surgery, Taizhou Central Hospital, Taizhou University, Taizhou, Zhejiang, China

**Keywords:** immune checkpoint inhibitors, gastroesophageal junction or gastric cancer, neutrophil to lymphocyte ratio, derived neutrophil to lymphocyte ratio, prognosis

## Abstract

**Objective:**

We carried out the meta-analysis to determine the predictive value of baseline neutrophil to lymphocyte ratio (NLR) and derived neutrophil to lymphocyte ratio (dNLR) levels in patients with gastroesophageal junction or gastric cancer (GJGC) who underwent immune checkpoint inhibitor (ICI) treatment.

**Methods:**

Eligible articles were obtained through PubMed, the Cochrane Library, EMBASE, and Google Scholar, until April 15, 2023. The clinical outcomes evaluated in this study encompassed overall survival (OS), progression-free survival (PFS), objective response rate (ORR), and disease control rate (DCR)

**Results:**

A total of 24 articles with 2221 patients were included in this meta-analysis. The pooled results demonstrated that patients with high NLR levels had significantly poorer OS (HR: 1.860, 95% CI: 1.564-2.213, *p* < 0.001) and PFS (HR: 1.678, 95% CI: 1.354-2.079, *p* < 0.001), and lower ORR (OR: 0.754, 95% CI: 0.621-0.915, *p* = 0.004) and DCR (OR: 0.391, 95% CI: 0.262-0.582, *p* < 0.001). Besides, we also found that high dNLR levels were significantly associated with shorter OS (HR: 2.117, 95% CI: 1.590-2.820, *p* < 0.001) and PFS (HR: 1.803, 95% CI: 1.415-2.297, *p* < 0.001).

**Conclusion:**

Low baseline (Derived) NLR has the potential to predict the good efficacy of ICIs and survival outcomes in patients with GJGC. (Derived) NLR could be useful in determining the optimal treatment strategies for these patients.

## 1 Introduction

Gastroesophageal junction or gastric cancer (GJGC) is one of the major causes of cancer-related mortality globally (1). Fluoropyrimidine plus a platinum agent is the most commonly used first-line therapy for patients with unresectable advanced or metastatic GJGC (2–4). Second-line chemotherapy, such as taxane with or without ramucirumab or irinotecan (2–4), is recommended for those who are refractory to such treatment. Despite significant advancements in chemotherapy and targeted therapies over the past few decades, the prognosis for patients with advanced GJGC remains unfavorable (5, 6). However, the emergence of immune checkpoint inhibitors (ICIs) has ushered in a new era of GJGC treatment. The monoclonal antibody against programmed death 1 (PD-1) has become a widely accepted standard of treatment for patients who have not responded to second-line or subsequent systemic therapies (7, 8). Nevertheless, due to limited efficacy and the possibility of severe toxicities, not all advanced GJGC patients are eligible for ICIs. Thus, the identification of predictive biomarkers that can distinguish advanced GJGC patients likely to have prolonged survival and tumor response to ICI therapy is crucial.

Numerous molecular and genomic biomarkers have been identified as predictive indicators for ICI therapy. These biomarkers include programmed death-ligand 1 (PD-L1) expression, tumor mutational burden, microsatellite instability status, and specific gene mutations (9–12). However, their clinical application is limited due to requirements for adequate tumor tissue and DNA sequencing and the absence of standardized quantitative scoring methods for PD-L1 immunohistochemistry (13, 14). This presents a need for easily accessible and cost-effective biomarkers that can be used in diverse settings, such as resource-poor areas, without relying on advanced genomic technologies or specialized expertise. Factors in the peripheral blood are potential candidates for such biomarkers.

The main focus of studies exploring immune-related markers in peripheral blood with respect to cancer has been on the neutrophil-to-lymphocyte ratio (NLR). NLR is calculated by dividing the absolute counts of neutrophils and lymphocytes and is thought to reflect the balance between the anti-tumoral immune response and the pro-tumoral inflammatory status. Numerous meta-analysis studies have demonstrated the value of NLR as a prognostic factor across a variety of cancers (15–17).

Nevertheless, there is disagreement regarding the predictive relationship between NLR levels and ICI-treated GJGC patients, and no pertinent meta-analysis has been carried out. Thus, in GJGC patients receiving ICI treatment, the prognostic value of NLR was comprehensively assessed in this investigation. This analysis could help in prognostication and treatment strategy development, leading to more precise, economical, and minimally harmful treatments.

## 2 Methods

### 2.1 Literature search strategies

The analysis conducted adhered to the PRISMA statement (18). On April 15, 2023, a thorough literature search was conducted on PubMed, EMBASE, and the Cochrane Library. A comprehensive range of search terms were used, including “Immune Checkpoint Inhibitors [Mesh]”, “Immune Checkpoint Blockers”, “Pembrolizumab”, “Nivolumab”, “Atezolizumab”, “Stomach Neoplasms [MeSH]”, “Stomach Cancer”, “Gastric Cancer”, “Neutrophil to Lymphocyte Ratio", “NLR”, “Derived Neutrophil to Lymphocyte Ratio", and “dNLR”. **Supplementary Table S1** has a thorough description of the search tactics. In addition to the primary database search, we conducted a supplementary search on Google Scholar to identify grey literature. Furthermore, we manually reviewed the reference lists of relevant articles to identify additional studies that may have been missed in the initial electronic search.

### 2.2 Inclusion and exclusion criteria

Only research publications that met the following criteria were considered for inclusion in our study: patients who had been diagnosed with GJGC were treated with ICIs, and the prognostic importance of NLR, also known as derived Neutrophil to Lymphocyte Ratio (dNLR), was evaluated. Moreover, the articles reported on at least one of the following endpoints: overall survival (OS), progression-free survival (PFS), objective response rate (ORR), and disease control rate (DCR). Studies that were excluded from the analysis included conference abstracts, editorials, case reports, and comments. In cases where there were patients who were included in more than one study, we chose only those studies that had the most comprehensive data and methodologies that were manually extracted (19).

### 2.3 Data extraction and quality assessment

We systematically extracted the following data from the eligible studies (1): Study characteristics: author(s), year of publication, study period, and region of study; (2) Participant characteristics: sample size, mean age, gender distribution; (3) Intervention details: therapeutic drugs used, cancer type, and cut-off values for NLR/dNLR; (4) Outcome measures: reported endpoints (OS, PFS, ORR, DCR), including hazard ratios (HR), odds ratios (OR), and confidence intervals (CI). For studies that included both univariate and multivariate analyses, we prioritized the extraction of multivariate data (HR and OR) as these analyses adjust for potential confounders and provide more reliable estimates. The quality of each observational study was assessed using the Newcastle-Ottawa Scale (NOS), with studies receiving a score of 6 or higher considered to be of excellent quality (20). This was calculated by examining three factors: method of patient selection, comparability of the study groups and number of outcomes reported. Two reviewers conducted literature search, literature screening, data extraction and literature quality assessment independently, and any disagreements were resolved by re-examining the relevant papers until a consensus was reached.

### 2.4 Statistical methods

This analysis was conducted using Stata 15.0. The chi-squared test was used to assess heterogeneity. A random effects model was employed when the *p*-value was less than 0.1 or the I^2^ statistic exceeded 50%; otherwise, a fixed effects model was used (21). The estimation of publication bias was conducted using the Egger and Begg tests. If a publishing bias exists, the "trim and fill" method was additionally employed to evaluate the influence on the combined outcomes. In addition, a sensitivity analysis was performed by systematically omitting each study to evaluate the strength and reliability of the findings.

## 3 Results

### 3.1 Characteristics of studies

**Figure 1:**
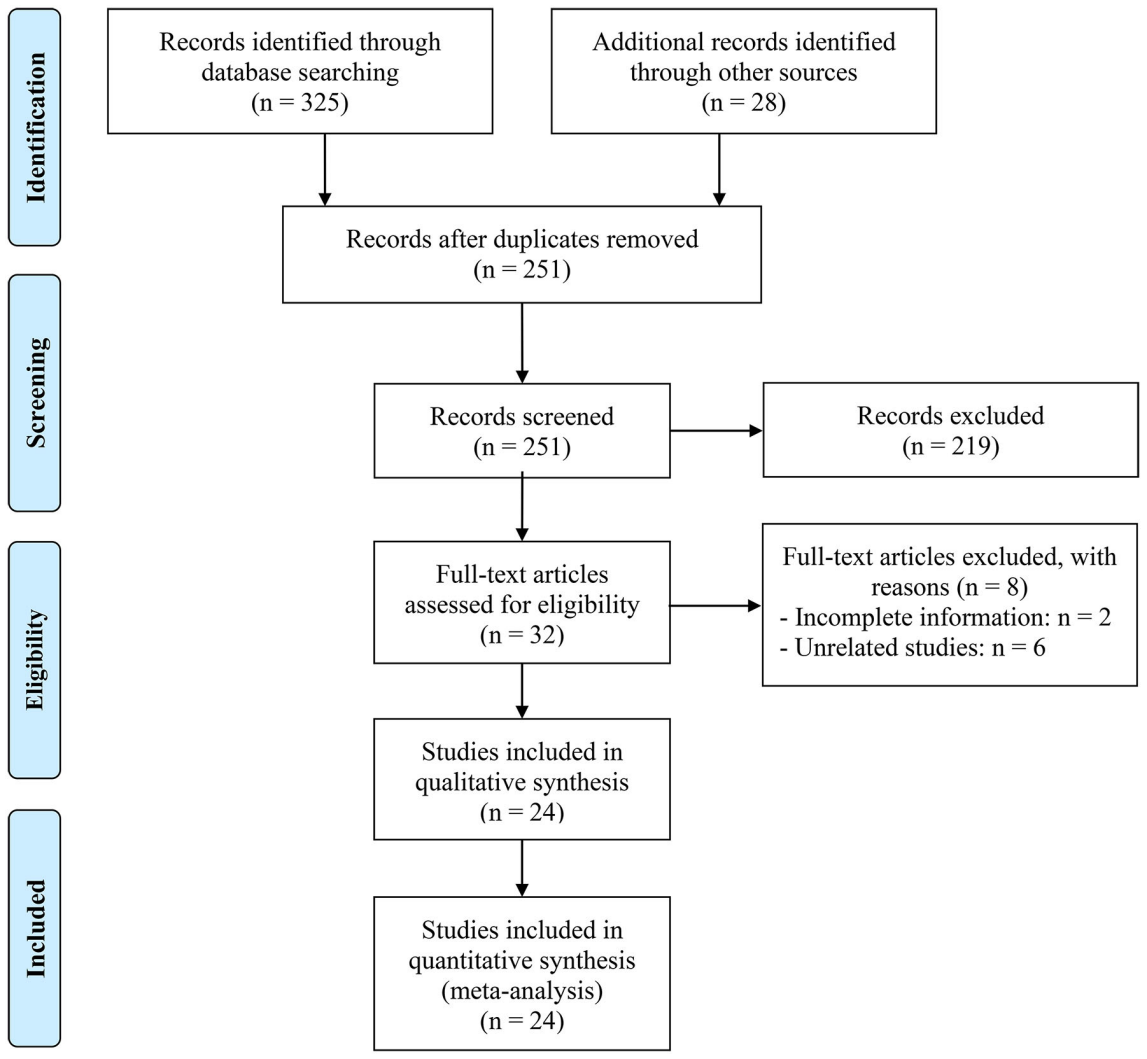
The flow diagram of identifying eligible studies.

After eliminating duplicates and carefully reviewing titles and abstracts, 32 articles were subjected to full-text examination, out of which 24 articles with a total of 2221 patients were selected for analysis (22–45). **Figure 1** shows the selection process using a PRISMA flow diagram. **Table 1** summarizes the main features of the included studies.

**Table 1:** Main characteristics of the studies included.

Study	Study design	Study period	Study region	Therapeutic drugs	Cancer type	Sample size	Age	Gender	Cut-off and outcomes	NOS
Hayano, et al., 2023 (42)	R	10/2018-12/2021	Japan	Nivolumab or Pembrolizumab	GC	70	71 (23-87)^a^	48/22	NLR=2.4 (OS, PFS)	7
Kawakami, et al., 2023 (43)	P	-	Japan	Nivolumab	GC	439	70 (26-90) ^a^	321/118	NLR=2.8 (OS, PFS)	8
Booka, et al., 2022 (35)	R	11/2017-12/2021	Japan	Nivolumab or Pembrolizumab	GC	30	71 (41-86)a	–	NLR=2.5 (OS)	6
Kim, et al., 2022 (36)	P	11/2014-02/2016	Korea	Nivoluma	G/GEJC	28	60 (23-76)^a^	21/7	NLR=2.9 (OS, PFS, ORR, DCR)	6
Li, et al., 2022 (37)	R	05/2015-05/2021	China	Nivolumab or Pembrolizumab	G/GEJC	54	11/43^c^	32/22	NLR=median (OS)	7
Sakai, et al., 2022 (38)	R	09/2017-03/2020	Japan	Nivolumab	GC	100	71 (38-89)^a^	78/22	NLR=2.5 (OS)	7
Tanaka, et al., 2022 (39)	P	10/2017-10/2019	Japan	Nivolumab	GC	70	69 (39-84)^a^	46/24	NLR=5.0 (OS)	7
Wan, et al., 2022 (40)	R	12/2017-06/2021	China	Camrelizumab, Sintilimab, Tislelizumab, Toripalimab or Envafolimab	G/GEJC	100	29/71^d^	77/23	NLR=3.9 (OS, PFS, ORR, DCR); dNLR2.45 (OS, PFS)	7
Xiang, et al., 2022 (41)	R	12/2020-04/2022	China	Camrelizumab	G/GEJC	49	19/30^c^	32/17	NLR=2.4 (PFS)	6
Gou, Et al. 2021 (28)	R	01/2016-11/2020	China	Nivolumab, Pembrolizumab, Sintilimab or Toripalimab	GC	137	81/56^d^	98/39	NLR=3.2 (OS, PFS, ORR, DCR)	7
Kim, et al., 2021 (29)	R	03/2016-06/2019	Korea	Pembrolizumab or Nivolumab	GC	185	59 (51-69)^b^	120/65	NLR=3.0 (OS, ORR, DCR)	8
Kubota, et al., 2021 (30)	R	10/2017-03/2019	Japan	Nivolumab	GC	26	65 (25-82)^a^	17/9	NLR=5.0 (PFS)	6
Pan, et al., 2022 (31)	R	12/2014-05/2021	China	Sintilimab, Toripalimab, Pembrolizumab or Nivolumab	GC	238	58 (18-86)^a^	176/62	dNLR=1.95 (OS, PFS)	8
Ruan, et al., 2021 (32)	P	12/2016-09/2017	China	Toripalimab	G/GEJC	58	60 (52-66)^a^	41/17	NLR=2.7 (OS, PFS, ORR, DCR)	6
Suzuki, et al., 2021 (33)	R	10/2017-02/2019	Japan	Nivolumab	GC	72	–	57/15	NLR=5.0 (OS)	7
Tokuyama, et al., 2021 (44)	R	02/2015-06/2019	Japan	Nivolumab	GC	45	65 (40–81)^a^	31/14	NLR=5.0 (OS)	7
Valero, et al., 2021 (34)	R	2015-2018	United States	ICIs	GC	71	34/37^d^	50/21	NLR=4.5 (OS, PFS, ORR, DCR)	6
Formica, et al., 2020 (23)	R	06/2014-12/2018	United Kingdom	Pembrolizumab, Nivolumab or Avelumab	G/GEJC	60	63 (33-87)^a^	43/17	NLR=Continuous (OS)	7
Li, et al., 2020 (24)	R	03/2016-04/2019	China	ICIs	GC	67	22/67^c^	43/24	NLR=3.0 (OS)	7
Namikawa, et al., 2020 (25)	R	10/2017-12/2019	Japan	Nivolumab	GC	29	71 (49-86)^a^	19/10	NLR=2.5 (OS, PFS)	6
Ota, et al., 2020 (26)	R	12/2014-09/2018	Japan	Nivolumab	GC	98	66 (33-84)^a^	68/30	NLR=3.0 (OS, PFS)	7
Yamada, et al., 2020 (27)	R	12/2014-02/2019	Japan	Nivolumab	GC	89	62/27^c^	42/47	NLR=2.5 (OS, PFS, ORR, DCR)	8
Kurosaki, et al., 2020 (45)	R	10/2017-03/2019	Japan	Nivolumab	G/GEJC	80	71(43-87)	67/13	NLR=5.0 (OS, PFS)	7
Ogata, et al., 2018 (22)	R	06/2017-12/2017	Japan	Nivolumab	GC	26	64 (44-86)^a^	19/7	NLR=5.0 (OS, PFS, ORR, DCR)	6

^a^medians (ranges); ^b^medians (interquartile range); ^c^≥ 65 vs. < 65; ^d^≥ 60 vs. < 60; R, retrospective study; P, prospective study; OS, overall survival; PFS, progression-free survival; ORR, objective response rate; DCR, disease control rate; G/GEJC, gastric or gastroesophageal junction cancer; GC, gastric cancer; ICIs, immune checkpoint inhibitors.

Of the 24 studies, 20 were retrospective studies and 4 were prospective studies. Seventeen included patients with GJGC, while seven included patients with gastroesophageal junction or gastric cancer. The low risk of bias was indicated by the NOS ratings for 24 publications, which ranged from 6 to 8.

### 3.2 Baseline NLR levels and OS

**Figure 2:**
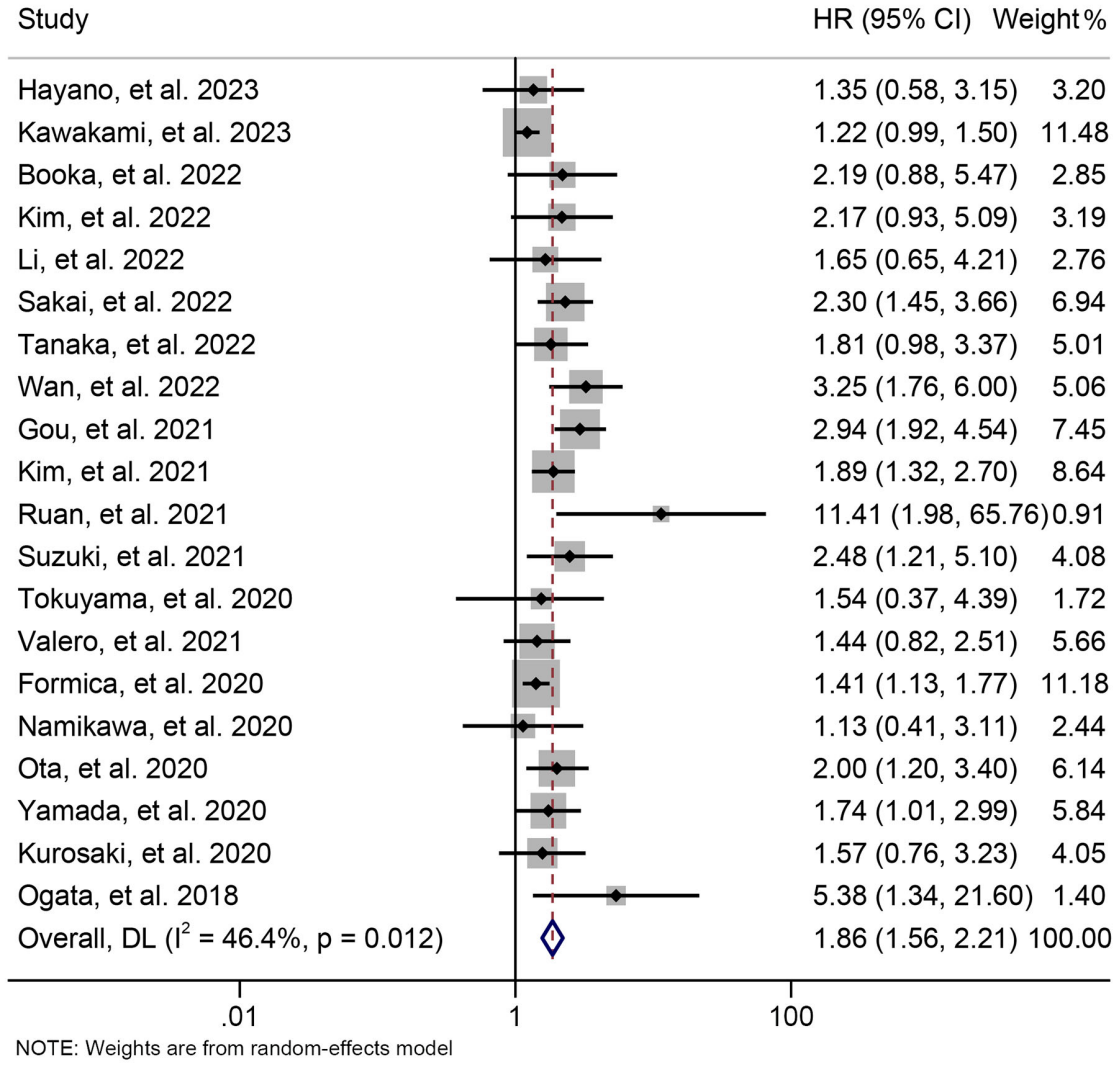
Forest plots of the relationship between neutrophil to lymphocyte ratio levels and overall survival. HR, hazard ratio; CL, confidence interval.

By examining the survival information from 20 trials with 1841 participants, we investigated the association between baseline NLR levels and OS in GJGC patients treated with ICIs. A random-effects model was utilized because of the significant heterogeneity among studies (I^2^ = 46.4%, *p* = 0.012). Our findings demonstrated that patients with high NLR levels had significantly worse OS (HR: 1.860, 95% CI: 1.564-2.213, *p* < 0.001) (**Figure 2**).

**Figure 3:**
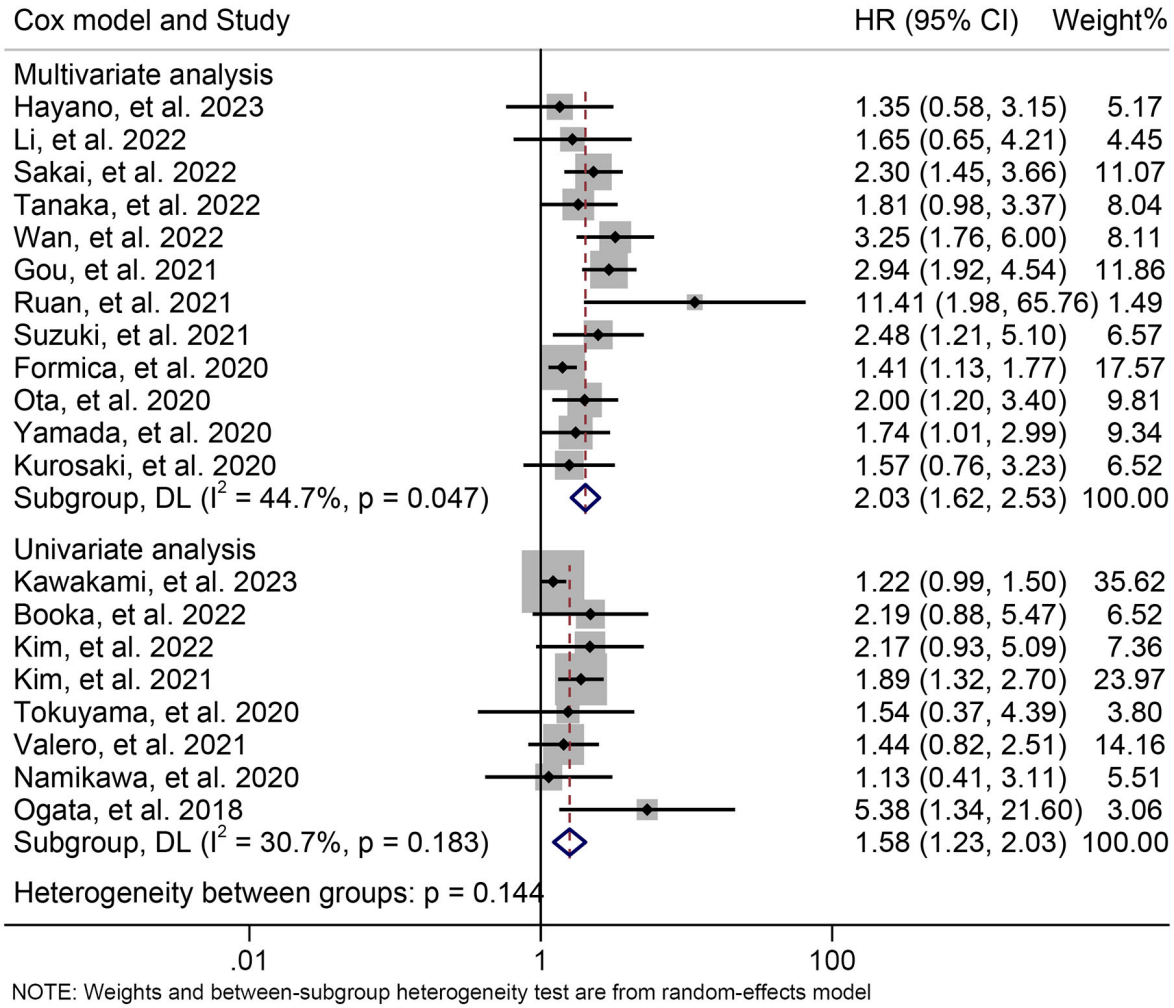
Subgroup analysis of the relationship between neutrophil to lymphocyte ratio levels and overall survival based on the Cox model. HR, hazard ratio; CL, confidence interval.

**Figure 4:**
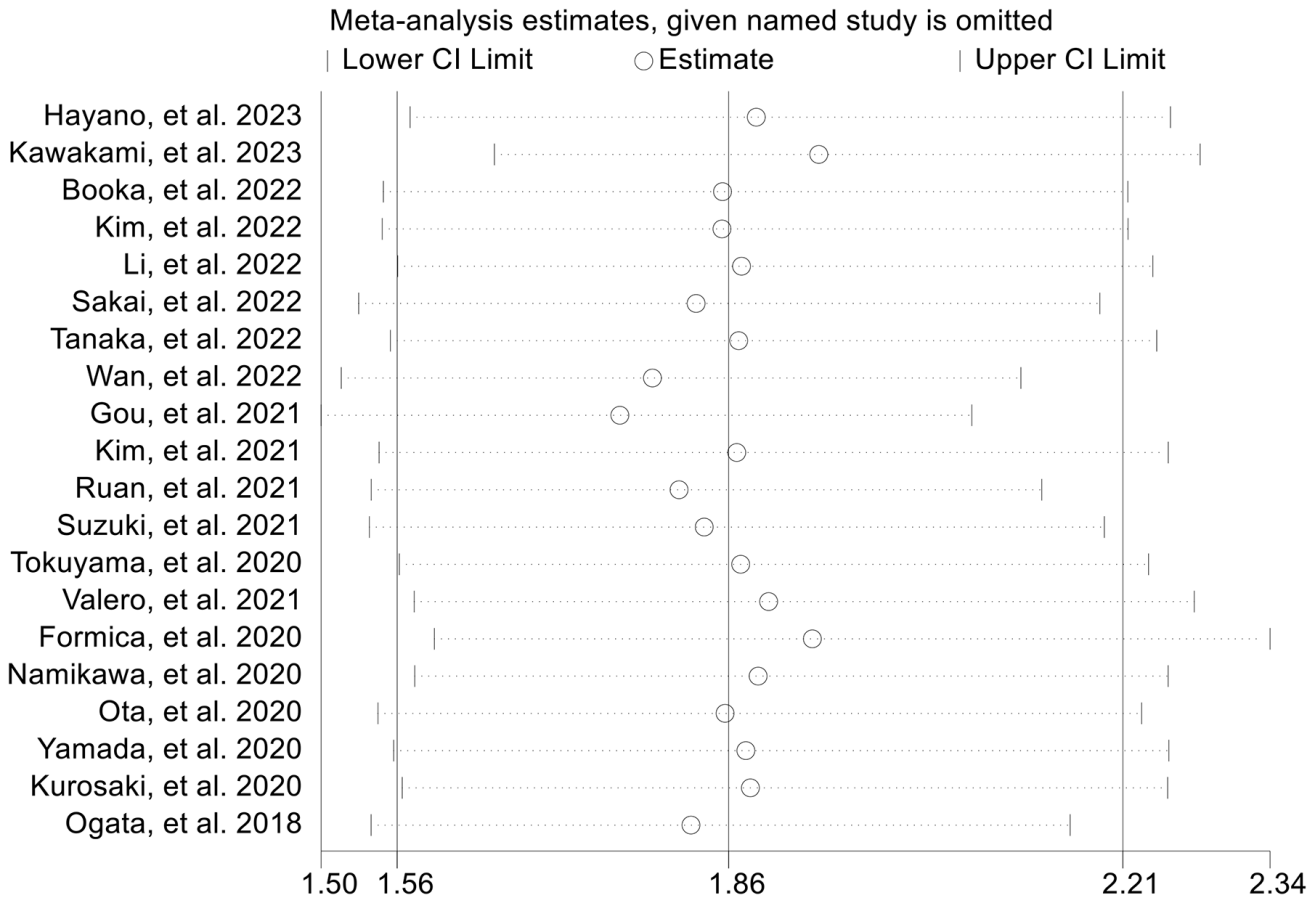
Sensitivity analysis of the association between baseline neutrophil to lymphocyte ratio levels and overall survival. CL, confidence interval.

The subgroup analysis was conducted based on the cut-off values and the Cox model. The above findings were consistent in each subgroup, as demonstrated in **Figure 3** and **Supplementary Figure S1**. Furthermore, a leave-one-out sensitivity analysis was carried out to evaluate the impact of each study on the overall results. The results indicated that the exclusion of any individual study did not significantly alter the pooled HR for OS, which ranged from 1.763 (95% CI: 1.496-2.078) after excluding Gou et al., 2021 to 1.935 (95% CI: 1.597-2.344) after excluding Formica et al., 2020 (**Figure 4**).

### 3.3 Baseline NLR levels and PFS

**Figure 5:**
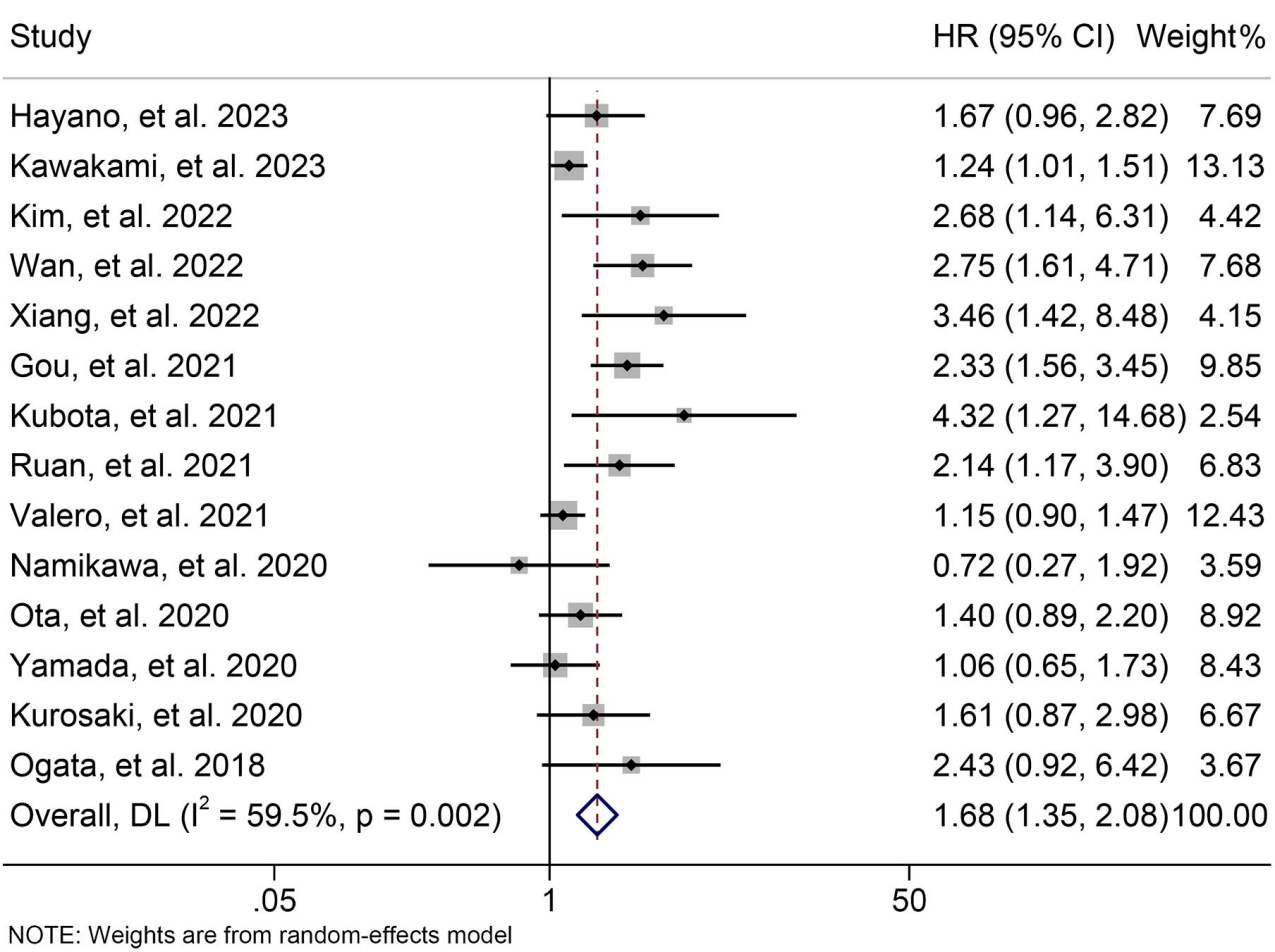
Forest plots of the relationship between neutrophil to lymphocyte ratio levels and progression-free survival. HR, hazard ratio; CL, confidence interval.

Using data from 14 studies involving 1300 patients, we examined the relationship between baseline NLR levels and PFS in ICI-treated GJGC patients. Our analysis revealed that high NLR levels were related to a 67.8% increase in the risk of progression (HR: 1.678, 95% CI: 1.354-2.079, *p* < 0.001, **Figure 5**). Due to significant heterogeneity (I^2^ = 59.5%, *p* = 0.002), we employed a random-effects model.

**Figure 6:**
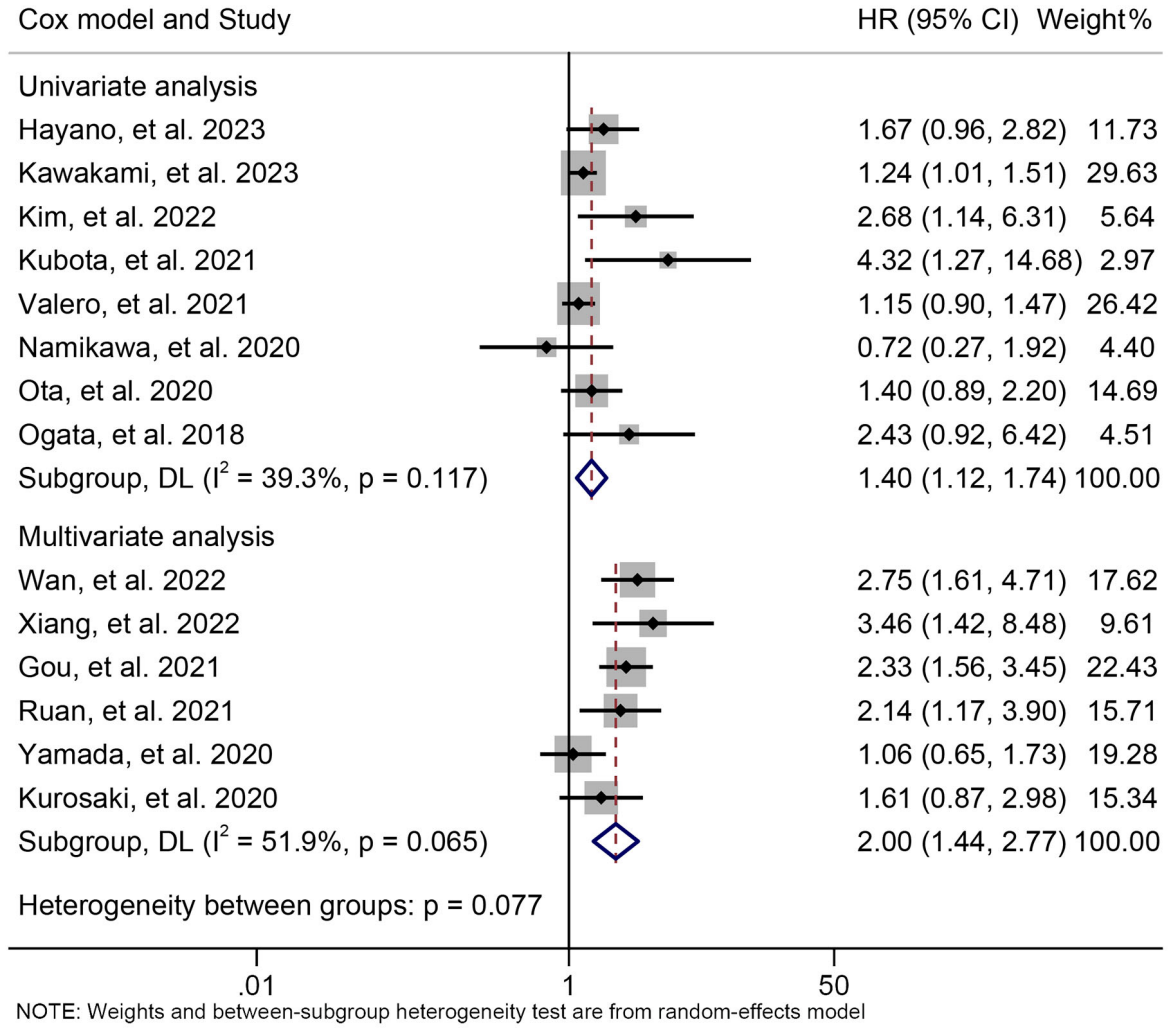
Subgroup analysis of the relationship between neutrophil to lymphocyte ratio levels and progression-free survival based on the Cox model. HR, hazard ratio; CL, confidence interval.

**Figure 7:**
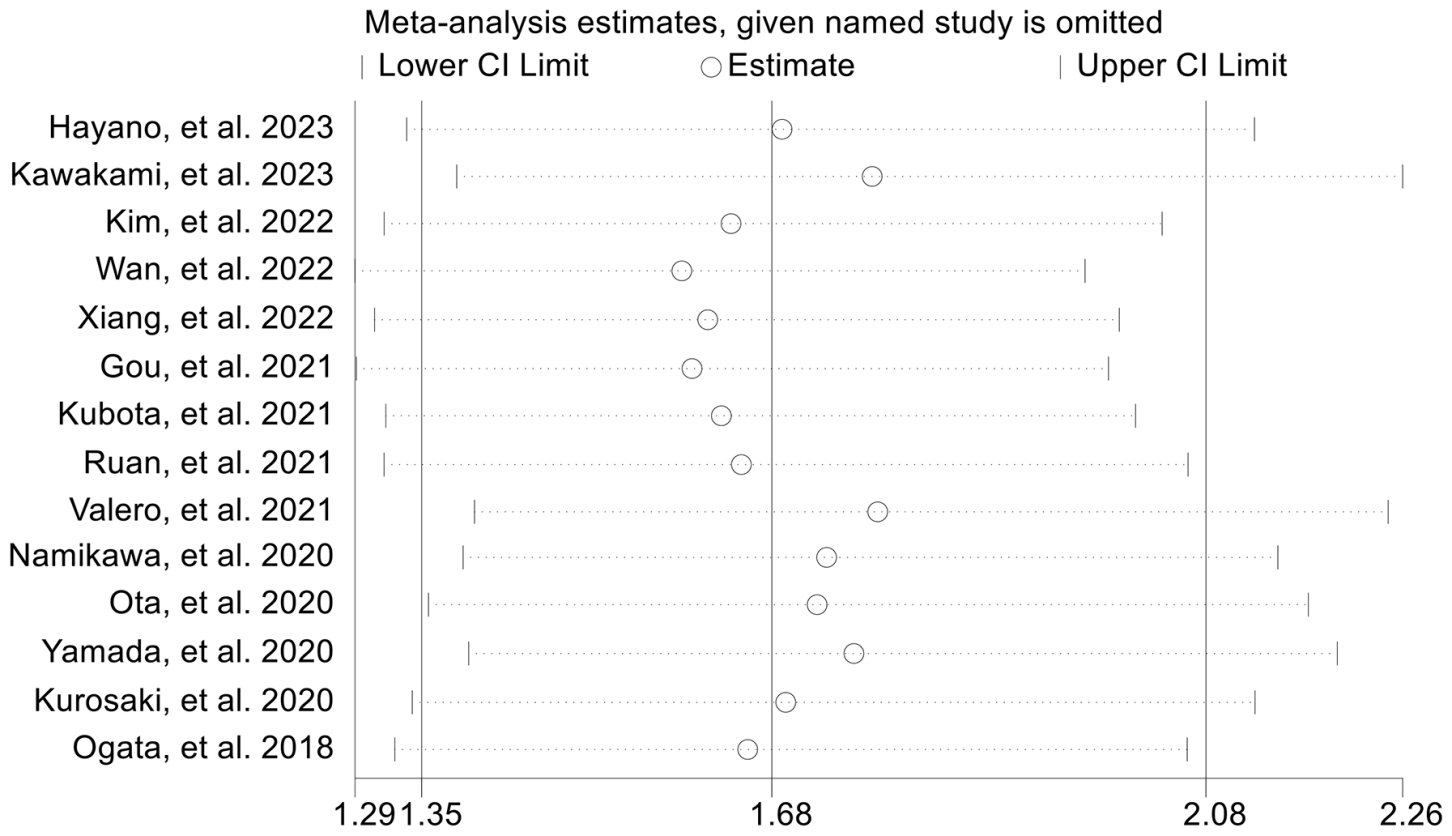
Sensitivity analysis of the association between baseline neutrophil to lymphocyte ratio levels and progression-free survival. CL, confidence interval.

The subgroup analysis demonstrated that the above findings hold for both multivariate and univariate analyses (**Figure 6**). Further subgroup analysis based on the cutoff value showed that high NLR levels were significantly linked to a shorter PFS when the cutoff value exceeded 2.5 (**Supplementary Figure S2**). However, we found no significant association between NLR levels and PFS when the cutoff value was ≤ 2.5 (**Supplementary Figure S2**). Moreover, the sensitivity analysis revealed that the exclusion of any single study did not result in a significant alteration of the pooled HR for PFS. The range of HR values was from 1.604 (95% CI: 1.294- 1.989) when removing Gou, et al., 2021 to 1.771 (95% CI: 1.387-2.261) when removing Kawakami, et al., 2023 (**Figure 7**).

### 3.4 Baseline NLR levels and ORR and DCR

**Figure 8:**
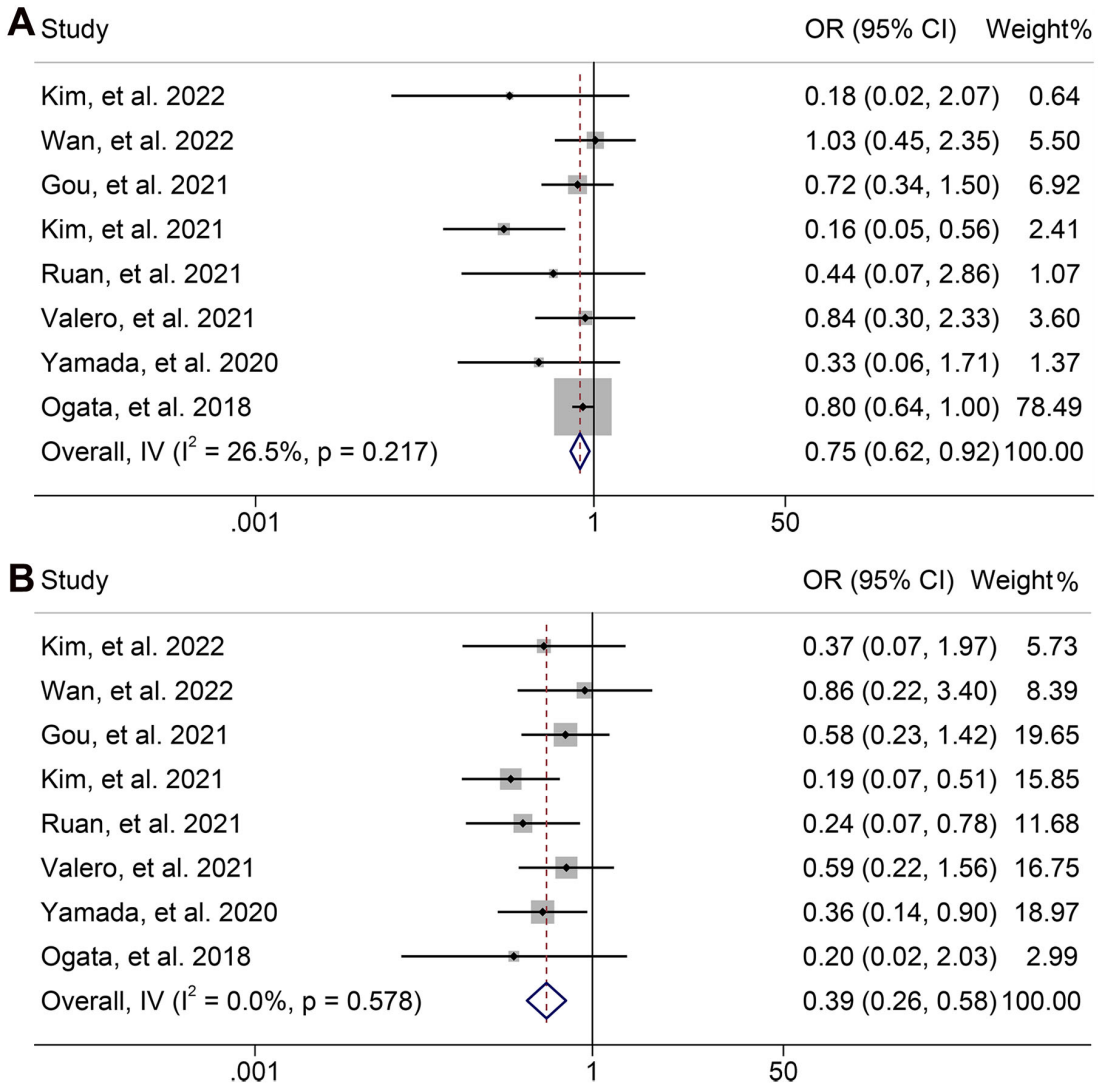
Forest plots of the relationship between neutrophil to lymphocyte ratio levels and objective response rate **(A)** and disease control rate **(B)**. OR, odds ratio; CL, confidence interval.

**Figure 9:**
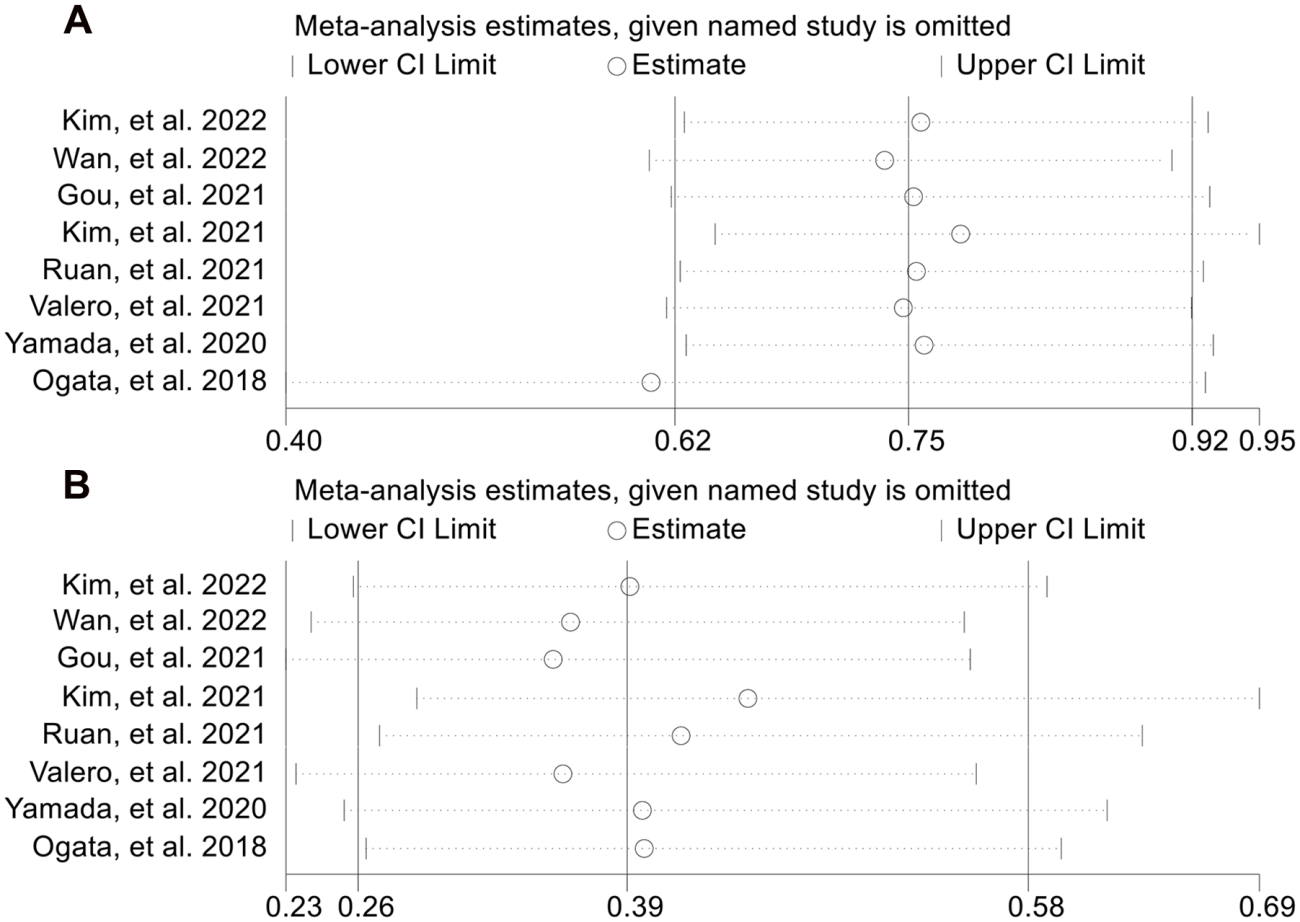
Sensitivity analysis of the association between baseline neutrophil to lymphocyte ratio levels and objective response rate **(A)** and disease control rate **(B)**. CL, confidence interval.

Next, we evaluated the relationship between NLR levels and response to ICI treatment in GJGC patients. As shown in **Figures 8A, B**, no significant heterogeneity was observed, so a fixed-effect model was used. We found that the ORR (8 studies with 694 patients, OR: 0.754, 95% CI: 0.621-0.915, *p* = 0.004, **Figure 8A**) and DCR (8 studies with 694 patients, OR: 0.391, 95% CI: 0.262-0.582, *p* < 0.001, **Figure 8B**) were lower in GJGC patients with high NLR levels. Besides, the pooled HRs for ORR (**Figure 9A**) and DCR (**Figure 9B**) were not significantly different in the sensitivity analysis.

### 3.5 Baseline dNLR levels and OS and PFS

**Figure 10:**
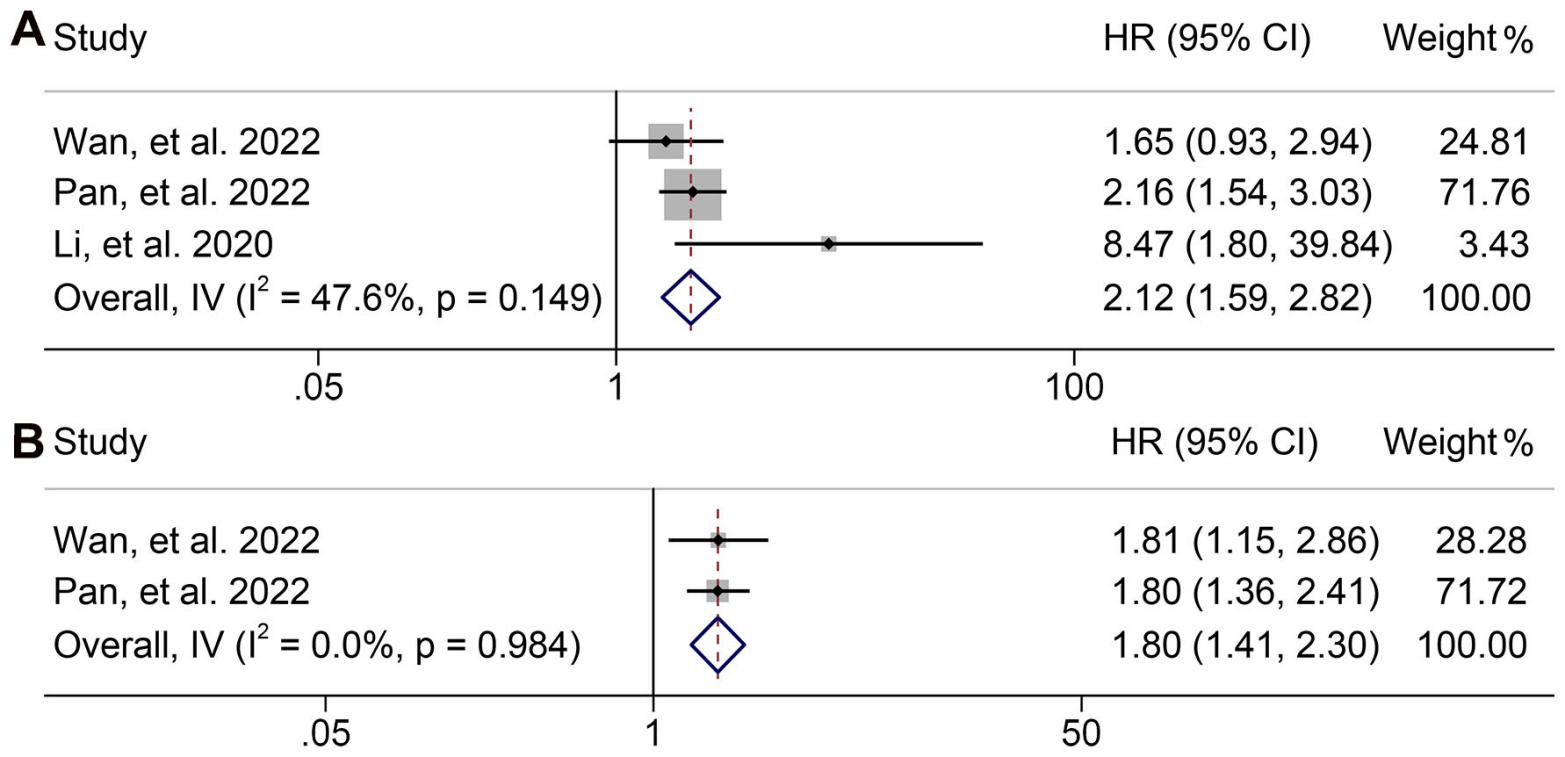
Forest plots of the relationship between derived neutrophil to lymphocyte ratio levels and overall survival **(A)** and progression-free survival **(B)**. HR, hazard ratio; CL, confidence interval.

A total of three articles comprising 405 patients and two studies involving 338 participants were included in the analysis to investigate the relationship between dNLR levels and OS and PFS in GJGC patients receiving ICI treatment, respectively. The pooled results revealed that high dNLR levels were significantly associated with shorter OS (HR: 2.117, 95% CI: 1.590-2.820, *p* < 0.001; I^2^ = 47.6%, *p* = 0.149, **Figure 10A**) and PFS (HR: 1.803, 95% CI: 1.415-2.297, *p* < 0.001; I^2^ = 0.0%, P = 0.984, **Figure 10B**).

### 3.6 Publication bias

We conducted Begg's and Egger's tests to evaluate potential publication bias in our analysis of NLR. The findings indicated the absence of significant publication bias concerning the ORR (Egger: *p* = 0.095, Begg: *p* = 0.073) and DCR (Egger: *p* = 0.727, Begg: *p* = 0.711). Nevertheless, in the assessment of OS (Egger: *p* = 0.006, Begg: *p* = 0.820) and PFS (Egger: *p* = 0.019, Begg: *p* = 0.228) using Egger's test, we identified evidence of publication bias.

To mitigate this concern, we applied the trim and fill method to estimate the potential number of omitted studies in OS and PFS. Following the incorporation of hypothetical missing studies, we recalculated the combined HR for OS (HR: 1.494, 95% CI: 1.237-1.805, *p* < 0.001) and PFS (HR: 1.393, 95% CI: 1.117-1.738, *p* < 0.001). Notably, this re-estimated value exhibited no significant deviation from the original result (see **Supplementary Figure S3**).

## 4 Discussion

Our study sought to examine the prognostic relevance of NLR in GJGC patients undergoing ICI treatment. Our meta-analysis confirmed a robust correlation between high NLR and inferior OS and PFS and lower ORR and DCR outcomes. Subgroup analysis revealed that NLR cutoff values greater than 2.5 had stronger predictive efficacy. In addition, we also confirmed that high dNLR levels were significantly related to shorter OS and PFS. Our study provide a comprehensive analysis of the influence of NLR or dNLR on the prognosis of ICI-treated GJGC patients. Given that NLR is a routine clinical parameter, evaluating its levels before ICI therapy could assist clinicians in predicting clinical outcomes more precisely and efficiently. This knowledge can facilitate timely adjustments to treatment plans and potentially enhance therapeutic benefits.

Kotecha et al. revealed an association between NLR and lymph node positive status in gastric cancer patients, which has important implications for staging and preoperative personalized treatment (46). Mellor et al. confirmed that NLR is an important prognostic indicator of OS and DFS after R0 resection of gastric cancer (47). Our study looked at ICI-treated patients with GJGC and confirmed the predictive value of NLR in such patients.

Cancer growth and progression are known to be characterized by ongoing inflammation and immune system evasion (48). The NLR has been suggested as a surrogate indicator of both inflammatory status and adaptive immune surveillance, reflecting the equilibrium between these two forces. The relationship between NLR and ICI outcomes may be explained by the association between circulating neutrophils and tumor microenvironment neutrophils (49–51).

Reactive oxygen species, matrix metalloproteinase 9, and vascular endothelial growth factor are examples of angiogenic and immunosuppressive mediators that can be produced by neutrophil infiltration and contribute to the development of a pro-tumor microenvironment (52–54). According to a study by Hiramatsu et al., there is a positive association between the NLR and the number of infiltrating tumor-associated neutrophils in the GJGC (55). Besides, Choi et al. confirmed that an elevated NLR is related to a reduced density of CD4^+^ lymphocytes in the tumor microenvironment (56). Furthermore, higher levels of myeloid cells, including neutrophils, may further suppress the T-cell response (57, 58). All of these elements combine to build an immunosuppressive tumor environment, which may lessen the likelihood that ICIs will work. Interestingly, recent studies suggest that neutrophils in the peripheral blood can interact with circulating tumor cells and enhance their metastatic potential by promoting cell cycle progression and accelerating metastasis seeding (59–62). These theories significantly support our finding that NLR levels can be a valid indicator of response to ICI in GJGC patients.

Proctor et al. have introduced the dNLR, which is calculated as the ratio of the neutrophil count to the difference between the white cell count and the neutrophil count (63). We also found that high dNLR levels also predicted a poor prognosis for ICI-treated GJGC patients. Then, the number of included studies was too small, and the finding needs to be further confirmed.

Our study provides preliminary evidence supporting the prognostic value of NLR and dNLR in predicting outcomes for ICI-treated GJGC patients. However, our findings also highlight areas where additional research is needed. First, as our results are largely based on data from retrospective studies and predominantly sourced from East Asian populations, it is crucial to conduct multicenter, prospective studies across diverse geographic and ethnic populations. This would help to validate our findings and improve their generalizability, ensuring they are applicable to a broader patient population. Additionally, future research should aim to identify the optimal NLR and dNLR cutoff values for clinical use, as these values may vary between different cancer subtypes and treatment regimens. Establishing standardized cutoff thresholds would facilitate the integration of NLR and dNLR measurements into routine clinical practice, providing a more consistent basis for treatment decision-making. Furthermore, mechanistic studies exploring the precise role of neutrophils and lymphocytes in the tumor microenvironment are warranted to elucidate the underlying biological pathways that link elevated NLR and dNLR with poorer ICI outcomes. Such insights could lead to targeted interventions aimed at modulating inflammatory and immune responses in the tumor microenvironment, potentially enhancing the efficacy of ICIs in patients with high NLR or dNLR.

In conclusion, our study underscores the prognostic relevance of NLR and dNLR in GJGC patients receiving ICI therapy, while also pointing toward critical avenues for future investigation. There is some heterogeneity in some results, so caution should be exercised in interpreting the conclusions. By addressing these areas, upcoming research can build on our findings, ultimately contributing to more precise and effective clinical management strategies for GJGC.

## Data Availability

The original contributions presented in the study are included in the article/**Supplementary Material**. Further inquiries can be directed to the corresponding author.

## Supplementary material

Supplementary Table S1 The detailed search strategies for Pubmed.

Supplementary Figure S1 Subgroup analysis of the relationship between neutrophil to lymphocyte ratio levels and overall survival based on cut-off. HR, hazard ratio; CL, confidence interval.

Supplementary Figure S2 Subgroup analysis of the relationship between neutrophil to lymphocyte ratio levels and progression-free survival based on cut-off. HR, hazard ratio; CL, confidence interval.

Supplementary Figure S3 The picture of the trim-and-fill method for OS **(A)** and PFS **(B)**.
