# Supplementary material for: Baseline (derived) neutrophil-lymphocyte ratio associated with survival in gastroesophageal junction or gastric cancer treated with ICIs
Source: Front Oncol. 2025 Jan 24;15:1404695. doi: 10.3389/fonc.2025.1404695 (PMC11802431; doi:10.3389/fonc.2025.1404695)
Supplement: Supplementary Table S1 — The detailed search strategies for Pubmed. [file Table1.docx]

Table S1. Pubmed search strategy

((((((((((((((((((("Neoplasm, Stomach"[Title/Abstract]) OR ("Stomach Neoplasm"[Title/Abstract])) OR ("Neoplasms, Stomach"[Title/Abstract])) OR ("Gastric Neoplasms"[Title/Abstract])) OR ("Gastric Neoplasm"[Title/Abstract])) OR ("Neoplasm, Gastric"[Title/Abstract])) OR ("Neoplasms, Gastric"[Title/Abstract])) OR ("Cancer of Stomach"[Title/Abstract])) OR ("Stomach Cancers"[Title/Abstract])) OR ("Gastric Cancer"[Title/Abstract])) OR ("Cancer, Gastric"[Title/Abstract])) OR ("Cancers, Gastric"[Title/Abstract])) OR ("Gastric Cancers"[Title/Abstract])) OR ("Stomach Cancer"[Title/Abstract])) OR ("Cancer, Stomach"[Title/Abstract])) OR ("Cancers, Stomach"[Title/Abstract])) OR ("Cancer of the Stomach"[Title/Abstract])) OR (Stomach Neoplasms[MeSH Terms])) AND (((((((((("neutrophil-to-lymphocyte ratio") OR ("neutrophil-lymphocyte ratio")) OR ("neutrophil to lymphocyte ratio")) OR ("neutrophil/lymphocyte ratio")) OR (NLR)) OR ("derived neutrophil to lymphocyte ratio")) OR ("derived neutrophil-to-lymphocyte ratio")) OR ("derived neutrophil-lymphocyte ratio")) OR ("derived neutrophil/lymphocyte ratio")) OR (dNLR))) AND (((((((((((((((((((((((((((((((((((((((((((((((((anti-CTLA4 antibody) OR (anti-CTLA4 antibodies)) OR (Immune Checkpoint Inhibitors)) OR (Checkpoint Inhibitors, Immune)) OR (Immune Checkpoint Inhibitor)) OR (Checkpoint Inhibitor, Immune)) OR (Immune Checkpoint Blockers)) OR (Checkpoint Blockers, Immune)) OR (Immune Checkpoint Blockade)) OR (Checkpoint Blockade, Immune)) OR (Immune Checkpoint Inhibition)) OR (Checkpoint Inhibition, Immune)) OR (PD-L1 Inhibitors)) OR (PD L1 Inhibitors)) OR (PD-L1 Inhibitor)) OR (PD L1 Inhibitor)) OR (Programmed Death-Ligand 1 Inhibitors)) OR (Programmed Death Ligand 1 Inhibitors)) OR (PD-1-PD-L1 Blockade)) OR (Blockade, PD-1-PD-L1)) OR (PD 1 PD L1 Blockade)) OR (CTLA-4 Inhibitors)) OR (CTLA 4 Inhibitors)) OR (CTLA-4 Inhibitor)) OR (CTLA 4 Inhibitor)) OR (Cytotoxic T-Lymphocyte-Associated Protein 4 Inhibitors)) OR (Cytotoxic T Lymphocyte Associated Protein 4 Inhibitors)) OR (Cytotoxic T-Lymphocyte-Associated Protein 4 Inhibitor)) OR (Cytotoxic T Lymphocyte Associated Protein 4 Inhibitor)) OR (PD-1 Inhibitors)) OR (PD-1 Inhibitor)) OR (PD 1 Inhibitors)) OR (Inhibitor, PD-1)) OR (PD 1 Inhibitor)) OR (Programmed Cell Death Protein 1 Inhibitor)) OR (Programmed Cell Death Protein 1 Inhibitors)) OR (pembrolizumab)) OR (nivolumab)) OR (atezolizumab)) OR (ipilimumab)) OR (avelumab)) OR (tremelimumab)) OR (durvalumab)) OR (cemiplimab)) OR (anti-PD-1 antibodies)) OR (anti-PD-1 antibody)) OR (anti-PD-L1 antibody)) OR (anti-PD-L1 antibodies)) OR (Immune Checkpoint Inhibitors[MeSH Terms]))
