# Supplementary figures and images for: Baseline (derived) neutrophil-lymphocyte ratio associated with survival in gastroesophageal junction or gastric cancer treated with ICIs
Source: Front Oncol. 2025 Jan 24;15:1404695. doi: 10.3389/fonc.2025.1404695 (PMC11802431; doi:10.3389/fonc.2025.1404695)

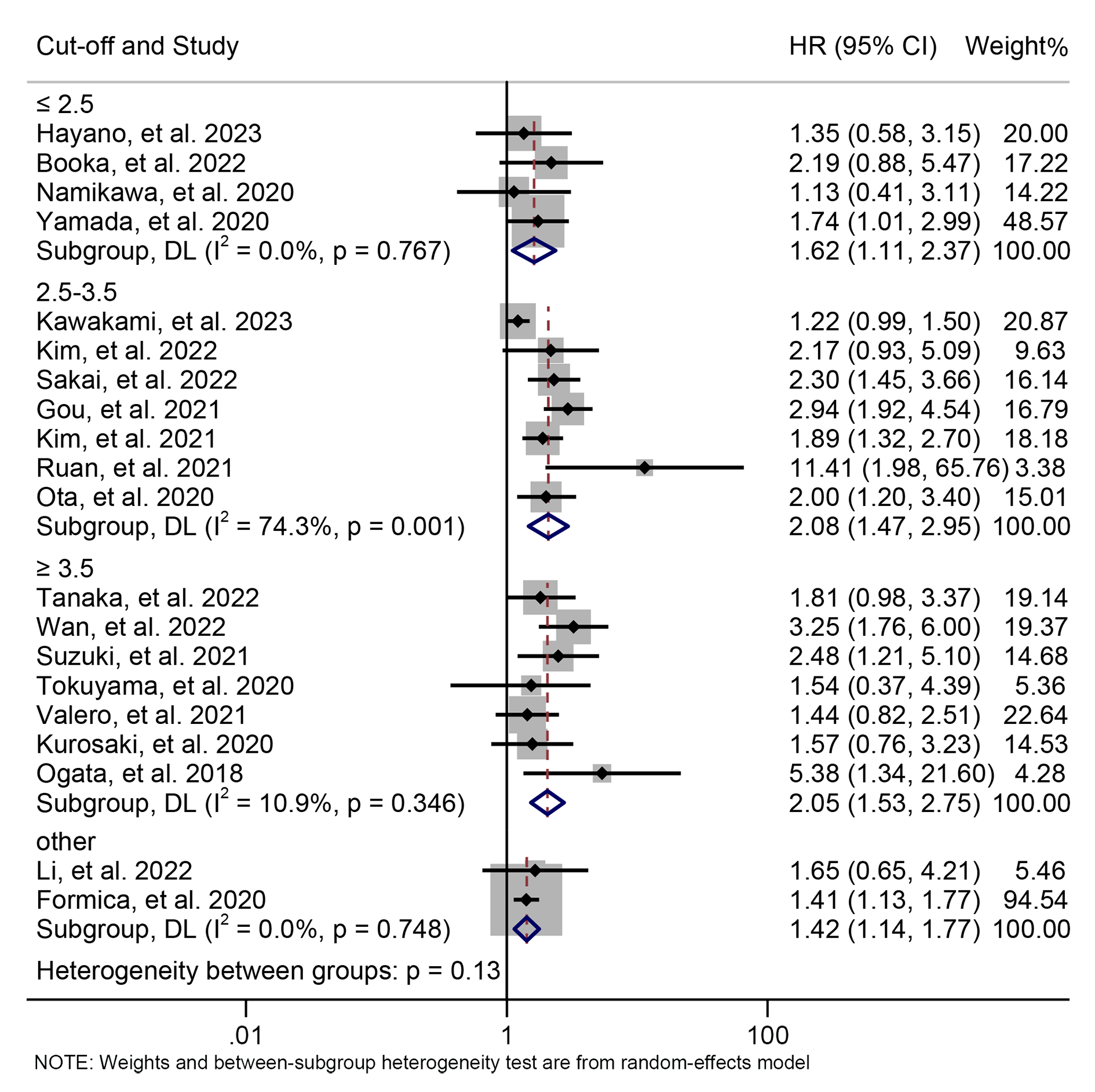

Supplement: Supplementary Figure S1 — Subgroup analysis of the relationship between neutrophil to lymphocyte ratio levels and overall survival based on cut-off. HR, hazard ratio; CL, confidence interval. [file Image1.tif]

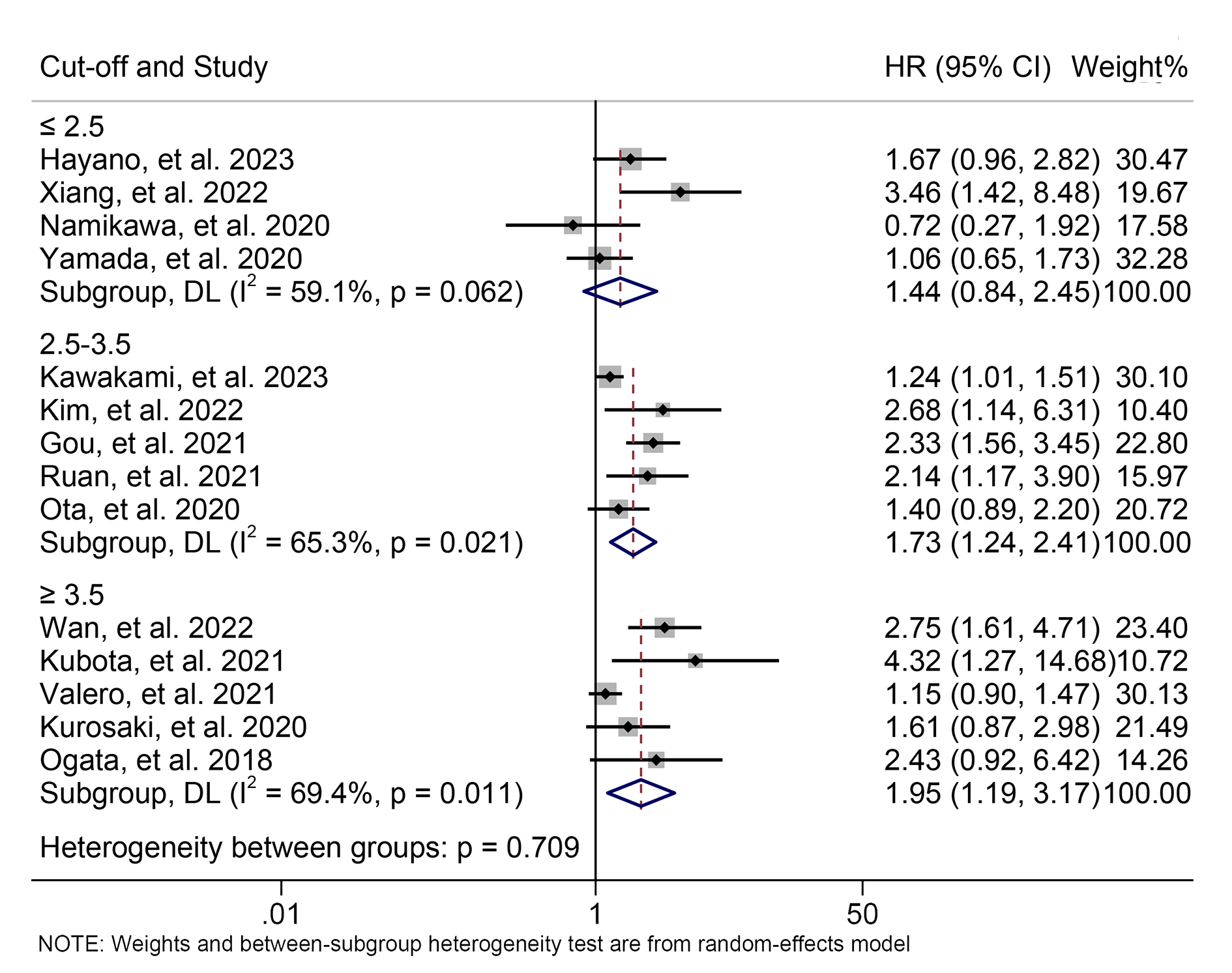

Supplement: Supplementary Figure S2 — Subgroup analysis of the relationship between neutrophil to lymphocyte ratio levels and progression-free survival based on cut-off. HR, hazard ratio; CL, confidence interval. [file Image2.tif]

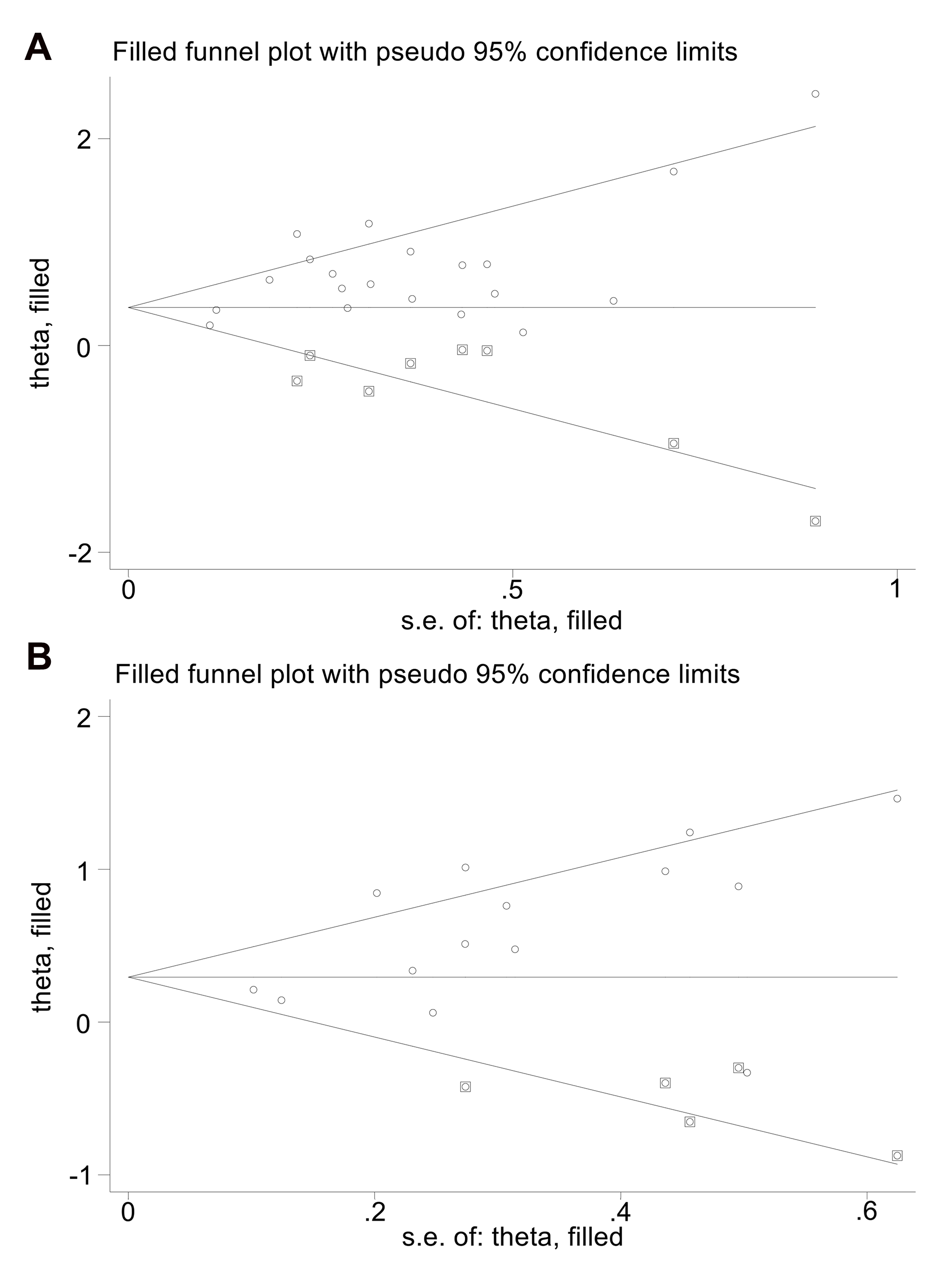

Supplement: Supplementary Figure S3 — The picture of the trim-and-fill method for OS (A) and PFS (B). [file Image3.tif]
